# The Association of Poor Preoperative Mental Health and Outcomes After Surgical Correction of Adult Spinal Deformity: A Systematic Review and Meta Analysis

**DOI:** 10.3390/jcm14155516

**Published:** 2025-08-05

**Authors:** Yifei Sun, Hariteja Ramapuram, Riyaz Razi, Mohammad Hamo, Sasha Howell, Nicholas M. B. Laskay, Jovanna Tracz, Anil Mahavadi, James Mooney, Jakub Godzik

**Affiliations:** 1 Heersink School of Medicine, University of Alabama at Birmingham, Birmingham, AL 35233, USA; ysun3@uab.edu (Y.S.); tejaram@uab.edu (H.R.); rrazi@uab.edu (R.R.); mahamo@uab.edu (M.H.); 2 Department of Neurosurgery, University of Alabama at Birmingham, Birmingham, AL 35233, USA; sghowell@uabmc.edu (S.H.); nl2991@cumc.columbia.edu (N.M.B.L.); jtracz@uabmc.edu (J.T.); akmahavadi@uabmc.edu (A.M.); 3 Department of Neurosurgery, Virginia Commonwealth University, Richmond, VA 23219, USA; mooneyj5@upmc.edu

**Keywords:** adult spinal deformity, mental health, depression, anxiety, outcomes, evidence-based medicine, systematic review, meta

## Abstract

**Background/Objectives:** Adult Spinal Deformity (ASD) is a pathologic malalignment of the spine that can lead to significant reductions in quality of life, functional limitations, and increased morbidity. While poor mental health is commonly observed among patients undergoing ASD surgery, its impact on surgical outcomes remains poorly understood. We conducted a systematic review and meta-analysis to examine the association between preoperative mental health and outcomes following surgical correction for ASD. **Methods:** A comprehensive search of MEDLINE, Embase, Web of Science, and Scopus was performed from inception to April 2025 to identify studies investigating the relationship between preoperative mental health and postoperative health-related quality of life outcomes or complications. Data was pooled using a restricted maximum likelihood (REML) random-effects model. Heterogeneity was assessed using Cochran’s Q statistic, and between-study variance was reported as τ^2^. Study quality was assessed with the Newcastle–Ottawa Scale, and risk of bias was evaluated using the ROBINS-I tool. **Results:** Twenty-four studies comprising a total of 248,427 patients met inclusion criteria. In pooled analyses, patients with poor preoperative mental health showed comparable improvements in health-related quality of life measures after surgery (standardized mean difference [SMD] −0.04, 95% CI −0.30 to 0.22; I^2^ = 91.5%, τ^2^ = 0.42) and in pain scores (SMD −0.15, 95% CI −0.42 to 0.11; I^2^ = 71.8%, τ^2^ = 0.09). However, patients with poor mental health had significantly higher odds of postoperative complications (odds ratio [OR] 1.44, 95% CI 1.23 to 1.67; I^2^ = 97.4%, τ^2^ = 0.08). These patients also demonstrated worse preoperative disease severity (SMD –0.94, 95% CI −1.41 to −0.47; I^2^ = 95.5%, τ^2^ = 1.64) and worse postoperative disease severity (SMD –0.34, 95% CI −0.44 to −0.25; I^2^ = 48.9%, τ^2^ = 0.03). **Conclusions:** While patients with poor preoperative mental health have a greater disease severity both before and after ASD surgery, they appear to experience comparable benefits from surgical intervention compared to those without. Recognizing and managing mental health may be useful in preoperative management of ASD patients. Further prospective studies to further elucidate these associations are necessary.

## 1. Introduction

Adult Spinal Deformity (ASD) is the pathologic malalignment of the spine and can result in a significant loss of quality of life, loss of function, and morbidity [1]. Often acquired from degenerative changes in the spine leading to significant sagittal misalignment or progressions from idiopathic deformities in adolescence, ASD presents an increasingly heavy burden on the healthcare system given our increasingly elderly demographic [2]. Significantly, ASD can often result in high rates of mental health comorbidities such as depression and self-image issues due to the deforming nature of these conditions [3,4]. Additionally, prolonged and chronic loss of function may also negatively affect mental health [5].

Surgical intervention is a highly effective method of treating ASD, resulting in an improved quality of life and function for many [6,7]. However, there has been increasing levels of evidence highlighting the role of preoperative mental health comorbidities in predicting postoperative outcomes in both the cervical and lumbar spine populations [8,9]. Several recent large studies and meta-analysis have emphasized the effect of depression, anxiety, and other mental health comorbidities in affecting health-related quality of life (HRQOL), pain, and complications in patients undergoing spinal surgery [8,9]. Significantly, the role of mental health comorbidities in the ASD population, which may be disproportionately enriched in mental health comorbidities, has been suggested by several studies [10,11,12]. However, the role of preoperative mental health in patients with ASD undergoing surgery, and whether it may affect benefits derived from surgical intervention, remains unclear.

Significantly, to our knowledge, no systematic review and meta-analysis have been conducted to ascertain the effects of preoperative mental health comorbidities on surgical outcomes in patients with ASD. We sought to perform a systematic review of the literature for any prospective, retrospective, or RCT studies that investigated the effect of preoperative mental health comorbidities, including but not limited to overall measures of mental health, depression, anxiety, bipolar, and PTSD, on surgical outcomes for ASD and to perform a meta-analysis of these results. In doing so, we hope to better understand the true effect of mental health comorbidities on ASD surgical outcomes.

## 2. Materials and Methods

We conducted a systematic review and meta-analysis according to a pre-determined protocol, which is registered at the International Platform of Registered Systematic Review and Meta-analysis Protocols (INPLASY) (registration number INPLASY202560113, Supplementary eAppendix 1). This manuscript was prepared in accordance with the Preferred Reporting Items for Systematic Reviews and Meta-Analysis (PRISMA) guidelines [13].

### 2.1. Search Strategy

We systematically queried PubMed/Medline, Embase, Web of Science, and Scopus from inception to April 2025. The search terms were key words to identify studies investigating preoperative mental health and adult spinal deformity. The full details of the search strategy can be found in Supplementary eAppendix 2.

### 2.2. Eligibility Criteria and Information Sources

Studies that discussed the association of preoperative mental health measured by any method, including preoperative mental health survey measures, depression, anxiety, PTSD, or any other mental health comorbidity with postoperative HRQOL or complications, were included. Studies that discussed pediatric or adolescent scoliosis or spinal deformities were excluded from analysis. Studies that did not provide sufficient granularity of data for analysis were excluded from analysis. Further details can be found in the supplemental digital content (Supplementary eAppendix 1).

### 2.3. Selection Process

Articles were screened for relevance and eligibility in the Covidence systematic review software (Veritas Health Innovation). After the removal of duplicate studies, two reviewers (YS, HR) independently screened the articles on the basis of abstracts and titles. After the initial screening, eligible articles were sought for full-text retrieval. At this stage, two reviewers (YS, HR) independently screened the articles for another round for relevance and inclusion. Any disagreements at any stage of selection between reviewers were resolved by consensus or with a third reviewer (JG).

### 2.4. Data Collection

Two investigators (YS, RR) extracted data from eligible studies in parallel using a standardized excel form. The data extracted was in accordance with the previously established protocol, including variables such as study characteristics, study design, demographic data of patients included, period of follow-up, mental health comorbidity assessed, method of assessment, and outcome.

### 2.5. Outcomes

The primary outcome for this meta-analysis was the change in HRQOL after surgery for ASD. Secondary outcomes of interest included complications and disease severity pre- and postoperatively.

### 2.6. Risk of Bias

A risk of bias analysis was conducted via two reviewers (YS, RR) who did not have an affiliation with the studies, using the Cochrane Risk-of-Bias in Non-randomized Studies of Interventions v2 (ROBINS-Iv2) tool (Supplementary eAppendix 3) [14]. Any disagreements were resolved via consensus or under advisement of a third reviewer (JG).

### 2.7. Study Quality

To evaluate study quality, we utilized the Newcastle–Ottawa scale (Supplementary eAppendix 4) [15]. Strength of evidence was assessed via the Grading of Recommendations, Assessment, Development, and Evaluations (GRADE) approach. The process was performed by one reviewer (YS) and agreed via consensus after review by other reviewers.

### 2.8. Data Synthesis

Results from the included studies were synthesized through meta-analysis to pool the effect sizes. The standard mean difference (SMD) was assessed for patient reported outcomes, and pooled OR was assessed for bivariate outcomes. Separate meta-analyses were performed to evaluate standardized mean differences (SMDs) in preoperative and postoperative disease severity between patients with and without poor mental health. Estimates were pooled using a restricted maximum likelihood (REML) random-effects model [16]. Between-group differences in preoperative severity were summarized using pooled SMDs, and postoperative outcomes were similarly assessed. Heterogeneity was quantified using the I^2^ statistic and between-study variance (τ^2^) [17]. Studies that were missing information were excluded from relevant analysis. Sensitivity analyses were performed via the leave-one-out method (Supplementary eAppendix 5) [18]. Publication bias was analyzed via the Egger test and visually assessed utilizing funnel pots (Supplementary eAppendix 6) [19]. To further assess potential sources of heterogeneity, we conducted meta-regression analyses to explore whether study-level characteristics accounted for variability in effect sizes. (Supplementary eAppendix 7–9) All statistical analyses were performed using R statistical software (version 4.3.1, R Foundation for Statistical Computing, Vienna, Austria) [20]. We utilized the meta package in the analysis [21]. The code used for analysis can be made available upon reasonable request.

## 3. Results

**Figure 1:**
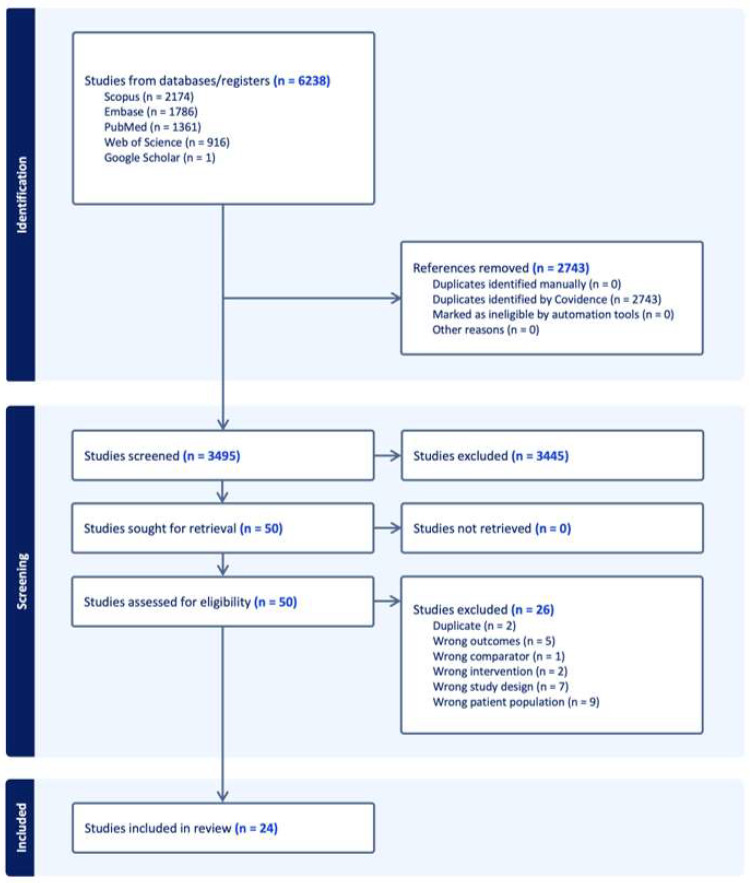
PRISMA flow diagram illustrating the study selection process for the meta-analysis.

A total of 6238 articles were identified, and 2743 articles were removed initially due to duplication. A total of 3495 articles underwent title and abstract screening. Of this, 3445 articles were excluded, primarily due to irrelevance. Of the 50 articles remaining that underwent full-text retrieval, 26 were excluded, primarily due to study population or study design. In total, 24 articles were included for final analysis (Figure 1) [10,11,12,22,23,24,25,26,27,28,29,30,31,32,33,34,35,36,37,38,39,40,41,42].

The study quality for these studies was assessed via the Newcastle–Ottawa scale (NOS). Of these studies, the majority (22/24) were of good quality. Risk of bias assessment via the ROBINS-I tool found overall risk of bias to be low to moderate overall.

A total of 248,427 patients were included across the 24 studies. Of all the studies, a large portion (10/24) were multicenter studies, with a few studies (3/24) utilizing large national datasets such as the national inpatient sample (NIS) or the PearlDiver database. The most common comorbidity assessed was depression or depression and anxiety (15/24). Amongst the studies, there were a variety of measures of mental health including ICD code-based, self-reported, or using patient reported forms such as the SF-36 Mental Component Score (SF-36 MCS) or the Scoliosis Research Society 22 (SRS-22) mental health domain. The details of the included studies can be found in Table 1.

**Figure 2:**
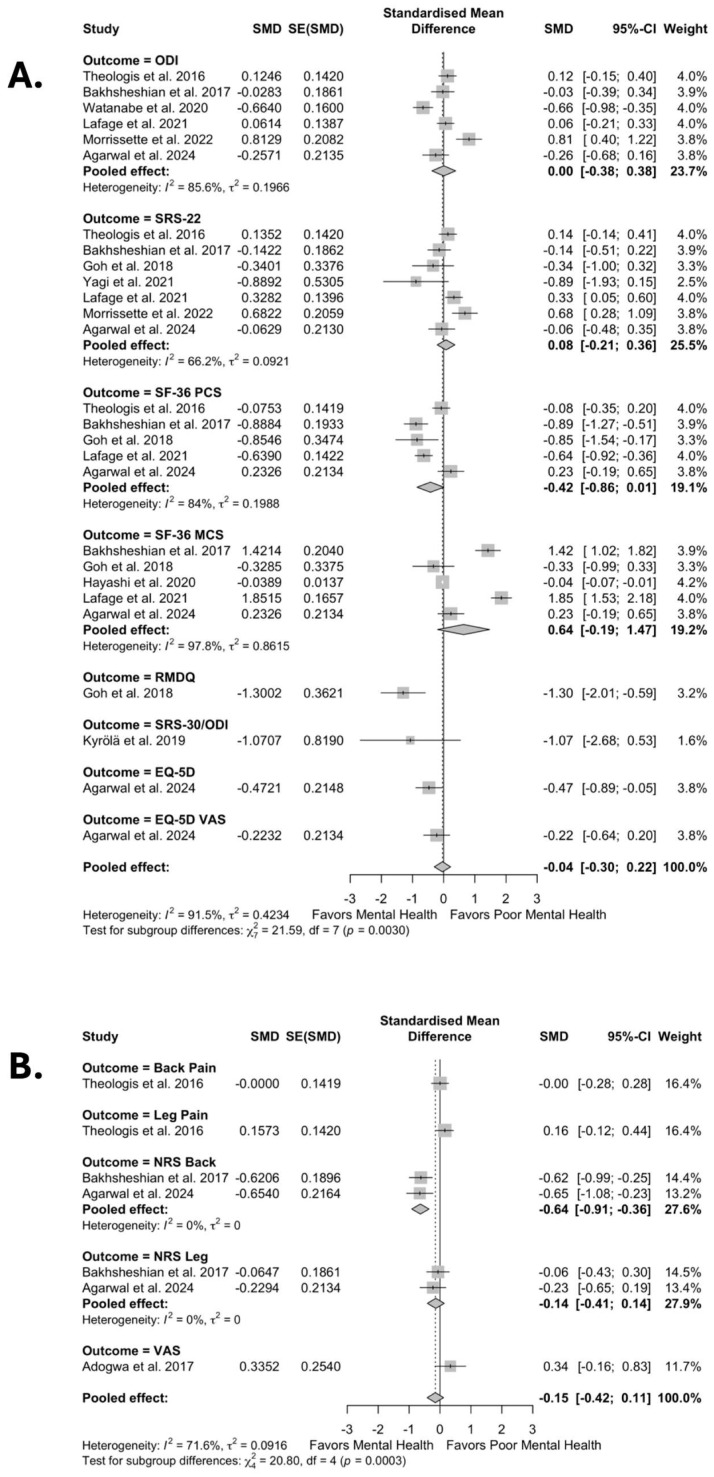
Forest plots for overall effect of mental health on improvement in (**A**) disability and function and (**B**) pain scores [10,11,12,22,26,27,29,30,33,39,41].

In pooled analyses, patients with poor preoperative mental health did not show significant differences in improvements in disability and functional measures after surgery (SMD –0.04, 95% CI –0.30 to 0.22; I^2^ = 91.5%, τ^2^ = 0.42) when compared to patients without poor preoperative mental health (Figure 2A).

**Figure 3:**
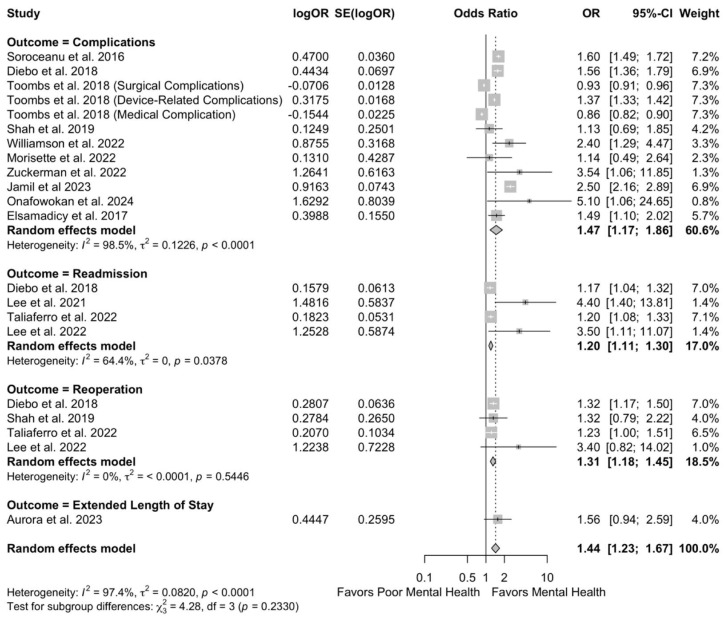
Forest plots for overall effect of mental health on complications [24,25,28,31,32,34,35,36,37,38,40,42].

Similarly, there were no significant differences in improvement in pain scores (SMD −0.15, 95% CI −0.42 to 0.11; I^2^ = 71.8%, τ^2^ = 0.09) after surgical intervention compared to patients without poor preoperative mental health (Figure 2B). However, patients with poor preoperative mental health had significantly higher odds of postoperative complications (OR 1.44, 95% CI 1.23 to 1.67; I^2^ = 97.4%, τ^2^ = 0.08) compared to patients without poor preoperative mental health (Figure 3).

**Figure 4:**
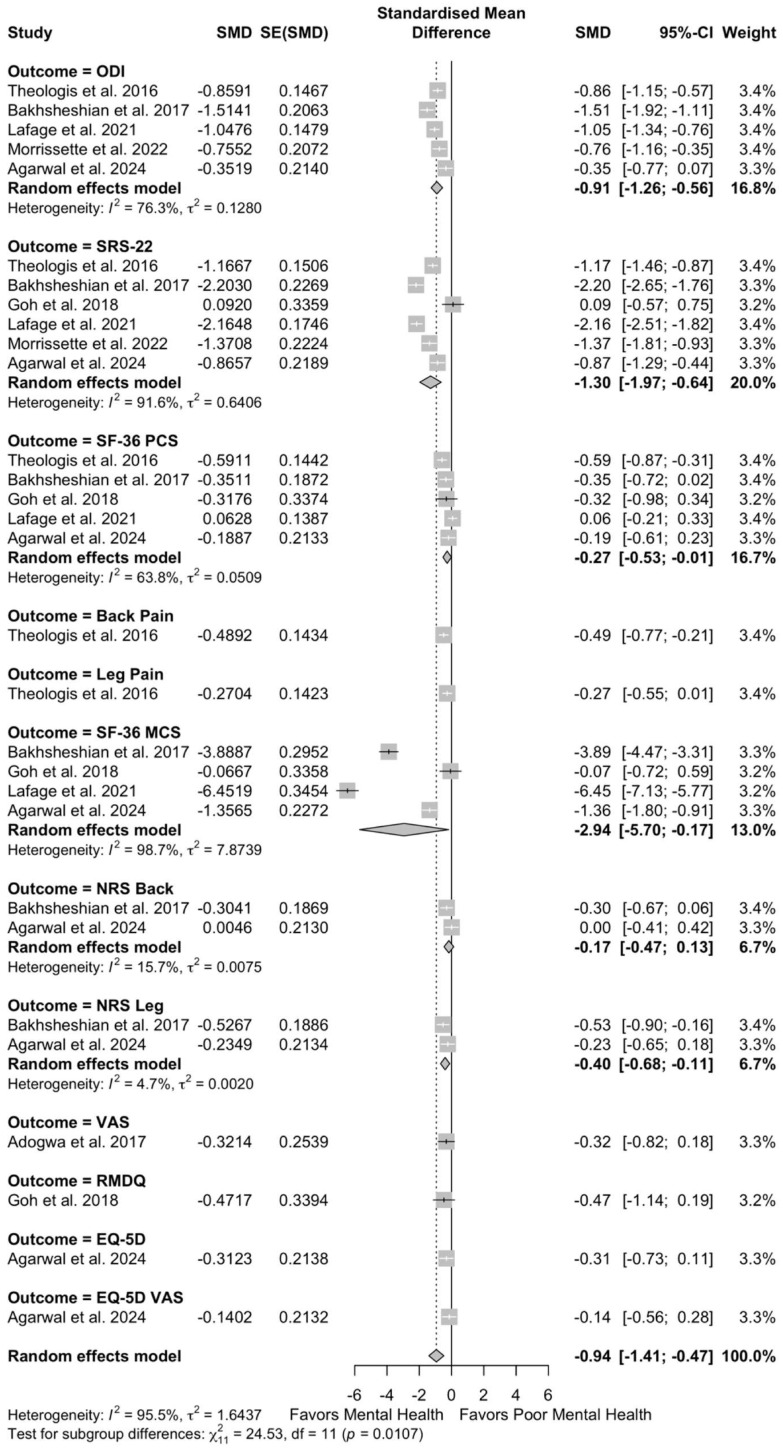
Forest plots for overall effect of mental health on preoperative disease severity [10,11,12,22,26,27,29,30,33,39,41].

**Figure 5:**
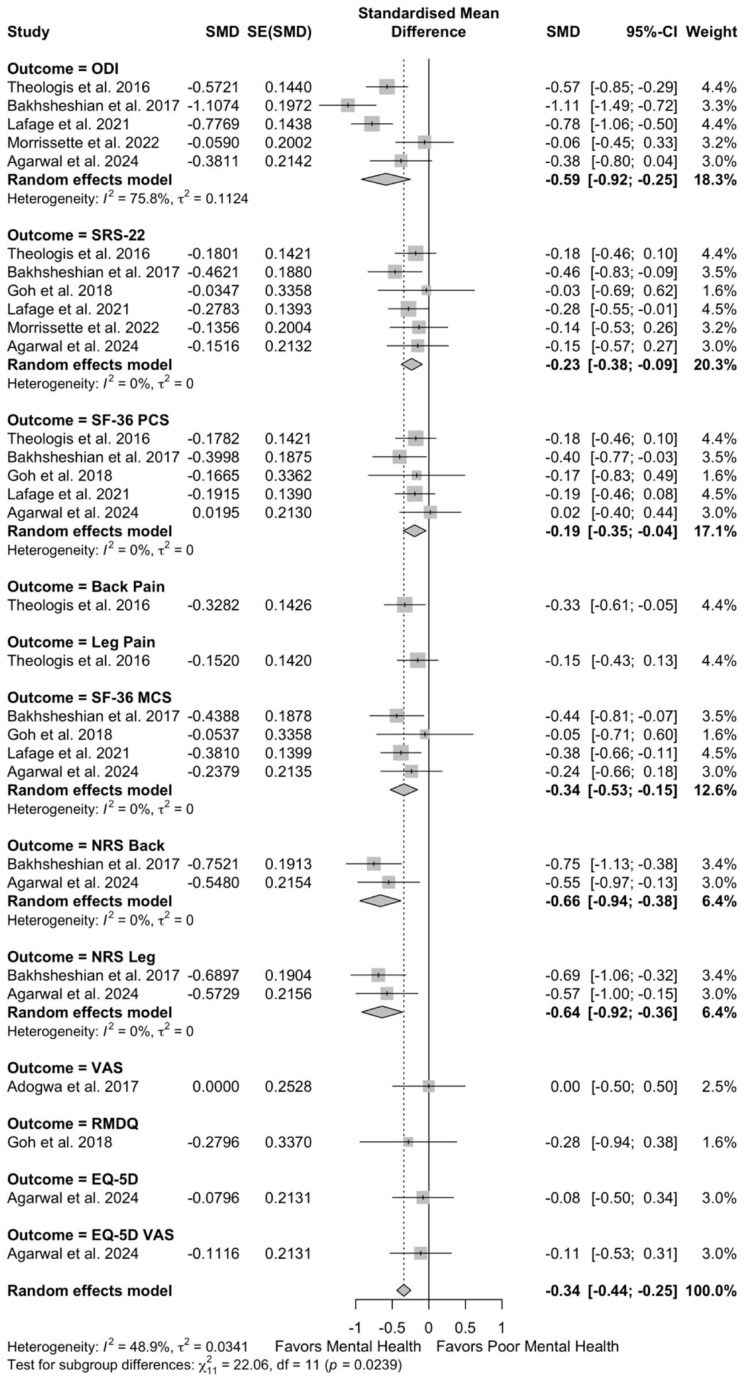
Forest plots for overall effect of mental health on postoperative disease severity [10,11,12,22,26,27,29,30,33,39,41].

These patients with poor preoperative mental health also demonstrated worse preoperative disease severity (SMD −0.94, 95% CI −1.41 to −0.47; I^2^ = 95.5%, τ^2^ = 1.64) and worse postoperative disease severity (SMD −0.34, 95% CI −0.44 to −0.25; I^2^ = 48.9%, τ^2^ = 0.03) (Figure 4 and Figure 5).

There was significant heterogeneity observed. To investigate the causes of heterogeneity across our primary models, we conducted a mixed-effects meta-regression to examine the influence of study-level covariates on effect sizes. In a meta-regression model evaluating predictors of effect sizes in studies reporting HRQOL outcomes, which accounted for 55.6% of the variance, we found that studies conducted in single-center settings (*p* <0.01), used the SF-36 MCS (*p* < 0.01) or SRS mental health domain (*p* < 0.01) as the measure of mental health, reported larger percentages of female patients (*p* < 0.01), and an increasing age in cohorts (*p* < 0.01) were associated with significantly larger effects compared to other studies. In a separate meta-regression model evaluating predictors of effect sizes in studies reporting pain outcomes, a greater proportion of female patients (*p* < 0.05) and increasing age (*p* < 0.05) were associated with increased effects. This model explained 55.5% of the variation observed. In a separate meta-regression model evaluating predictors of effect sizes in studies reporting complications, studies conducted in single-center settings were significantly associated with larger effect sizes compared to other settings (*p* < 0.05). This model explained 55.5% of the variation observed. Further details pertaining to this analysis can be found in Supplementary eAppendix 7–9. To additionally investigate sources of heterogeneity and to serve as additional sensitivity analysis, we investigated primary outcomes stratified by type of mental health comorbidity. The results held constant from previous analysis (Supplementary eAppendix 10,11).

After an assessment of the strength of evidence, all outcomes received an evaluation of moderate strength of evidence, with considerations for the lack of publication bias and for inconsistency in the assessment of type of mental health comorbidity (Supplementary eAppendix 12).

## 4. Discussion

As the U.S. population continues to age, the prevalence of adult spinal deformity (ASD) is expected to rise [2]. While surgical intervention has been shown to significantly improve HRQOL, it remains critical to identify perioperative factors that may influence surgical outcomes [6,7]. There has been additional focus on the effect of mental health comorbidities in ASD surgical outcomes [10,43]. This is particularly important due to the disproportionately higher rates of mental health conditions in patients affected by ASD, with studies highlighting self-image issues, depression, and anxiety [44,45]. However, current data remains conflicting as to the role of preoperative mental health comorbidities in determining surgical outcomes following surgical correction. Our study, which pools analyses from 24 studies, found that while lower levels of preoperative mental health was associated with worse functional and quality of life before and after surgical intervention compared to patients with good mental health, these patients derive similar benefits from surgical intervention in terms of improved HRQOL measures compared to patients without mental health comorbidities.

These results suggest that despite worse absolute preoperative disability, surgical intervention remains a viable option for patients with poor preoperative mental health. These findings can be attributed to the fact that HRQOL gains following ASD surgery are driven primarily by structural correction, pain relief, and restoration of function—outcomes that can directly benefit patients regardless of preoperative mental health status [44,46]. These physical improvements may have downstream positive effects on mood and psychosocial wellbeing, particularly in patients who experienced mental health distress due to chronic pain, deformity, or reduced mobility due to ASD [11,47]. Consequently, even patients with low baseline mental health may experience meaningful gains in both function and perceived quality of life, resulting in similar benefits derived from surgery when compared to those with better mental health at baseline.

Our results also found that poor preoperative mental health was associated with greater disease severity both before and after surgery. These findings may be reciprocal in nature: patients with poor mental health may experience psychological distress because of chronic pain, deformity, and reduced functional status, while in turn, their mental health symptoms, such as low self-efficacy, increased pain sensitivity, and avoidance behaviors, may delay care-seeking and impair rehabilitation [48]. This cyclical interaction can lead to sustained worse disease severity. As a result, patients with poor mental health may present with more advanced disease and continue to report worse postoperative status compared to patients without mental health comorbidities [9]. Importantly, however, these findings are consistent with our primary analysis, which demonstrated that despite these baseline differences, patients with poor preoperative mental health derive a similar relative benefit from surgery in terms of HRQOL improvement. Certain global measures of health-related quality of life may also have mental health components included as a part of the measure, which may correlate with their functional status and artificially inflate disease severity stratification by mental health status.

We also found that patients with poor preoperative mental health were at an increased risk of postoperative complications. Several mechanisms may contribute to this association. First, mental health disorders are often associated with lower adherence to discharge instructions, reduced engagement in postoperative care, and impaired self-management, all of which can increase the risk of complications [49]. Second, chronic mental health conditions are linked to elevated levels of pro-inflammatory cytokines. The association of inflammation induced by poor mental health is well established, as psychiatric conditions are often associated with elevations in systemic inflammatory markers [50,51]. These inflammatory markers including interleukin-1 (IL-1), interleukin-6 (IL-6), and tumor necrosis factor-alpha (TNF-α), which have been implicated in the pathophysiology of depression, anxiety, and chronic stress, may be an underlying contributor to complications by interfering with normal wound healing processes [50,52,53]. These chronic pro-inflammatory states that have been shown to impair wound healing may predispose patients to complications after surgery [54,55,56].

Furthermore, patients with poor mental health may be delayed in their presentation, necessitating more complex and extensive surgical interventions—procedures that inherently carry higher risks. This is consistent with our finding that patients with mental health comorbidities had greater preoperative disease severity, which may partially explain their elevated complication rates.

### 4.1. Management

Management of perioperative poor mental health may be necessary for patients with this risk factor, though strategies and approaches may differ based on pathology. Screening for mental health may differ based on targeted pathology, including using the PHQ-9 for depression, GAD-7 for anxiety, and MSC-36 mental health component to assess overall mental health status [57,58,59]. Though psychiatric counseling and medication management have been shown to effectively ameliorate symptoms of depression or anxiety, consideration of long-term systemic pathologies will be important to consider [60,61]. Chronic elevations of cortisol in patients with depression and anxiety will weaken immune responses and impair healing, necessitating an increased vigilance in wound care protocols and surveillance for dehiscence or infection [50,52,53].

Differences in pathology between depression and anxiety may require different interview strategies pre- and postoperatively. Patients with anxiety may require additional prophylactic sedative therapy, and approaches in counseling may target anticipatory anxiety or hypervigilance towards pain [62,63]. Additional motivational counseling and SSRI optimization may be more of a focus for patients with optimization [64]. For patients with poor preoperative mental health, undisclosed substance use disorder (SUD) may be a point of careful consideration for postoperative pain management, as SUD is a common comorbidity in patients with mental health comorbidities [65]. Large studies have suggested that patients with comorbid mental health conditions, particularly anxiety more than depression, are less likely to be benzodiazepine naïve, and will therefore react strongly with an increased risk of side effects to various classes of sedatives [66]. Additionally, careful titration of SSRI levels in the weeks leading up to and after surgery may be important to optimize bone healing and fusion, particularly in ASD correction operations [67,68].

Recognition of underlying, commonly misdiagnosed personality disorders preoperatively may be crucial for optimizing postoperative care, as mood swings and nonadherence associated with Cluster B and Cluster C personality disorders may complicate the ability to deliver quality care [69]. Early referral and coordination with a multidisciplinary surgical-psychiatric care team may be ideal in caring for these patients. Taken all together, the various complexities involved in the care of patients with mental health comorbidities include the recognition of an increased risk of acute psychotic events postoperatively, increased risk of delirious events, potential for long term abuse of substances due to lower pain tolerance thresholds, and increased potential for abuse [25,65,70,71].

Postoperatively, outpatient referrals may be necessary for patients with diagnosed mental health comorbidities to improve adherence to postoperative care plans and rehabilitation [72,73].

Our findings additionally strongly highlight the need for the standardization of mental health assessments and measures. Across the studies, the survey utilized to measure the mental health comorbidity was seldom reported, primarily being described as “self-reported” or previously diagnosed/ICD code-based (Table 1). While certain study designs make these decisions best from a logistical standpoint, the standardization of measures of depression, anxiety, and other mental health comorbidities may be necessary for the increased generalizability of findings and greater agreement between studies.

In our meta-regression analysis, we found that studies with older patient populations reported larger effect sizes for the impact of poor preoperative mental health on postoperative outcomes. These findings suggest that older adults may be more sensitive to the adverse effects of psychological distress. Advanced age is often associated with increased frailty, a higher burden of medical comorbidities, reduced physiological reserve, and a diminished capacity for recovery—all of which may amplify the impact of poor mental health on surgical outcomes [74,75,76,77]. As such, targeted preoperative mental health optimization may be particularly important in older patients to mitigate risk and enhance postoperative recovery.

### 4.2. Limitations

This study has several limitations. First, although many of the included studies were multicenter in design, all were retrospective observational studies. This limits the strength of causal inferences that can be drawn from our meta-analysis. Second, the limited granularity of available data precluded a patient-level pooled analysis, which may have yielded more precise and nuanced findings. Several studies relied on national databases, which often lack radiographic measures such as sagittal and coronal balance parameters, core metrics used to define ASD severity, thereby limiting interpretability. Residual confounding remains possible, as not all studies adjusted for key clinical or psychosocial variables that may influence both mental health and surgical outcomes. Differences in surgical approach across centers and evolving techniques over time may also contribute to the heterogeneity observed. Differences in surgical complexity, which were not consistently reported or adjusted for, may have influenced outcomes and contributed to heterogeneity. Although there remains the possibility of publication bias due to unpublished negative studies, our assessment suggests this risk is low. There was heterogeneity in how mental health was assessed across studies, ranging from patient-reported outcomes to diagnostic codes, which may limit cross-study comparability and introduce misclassification bias. There is additional heterogeneity from unmeasured and unreported confounders and differences in study planning and setting that may contribute to additional heterogeneity. While significant statistical heterogeneity was present, we conducted rigorous sensitivity analyses and meta-regressions to explore and account for potential sources of variation between studies, and our models were able to explain a substantial amount of the heterogeneity, though some persisted. Despite these limitations, this study represents the first comprehensive meta-analysis examining the impact of preoperative mental health on surgical outcomes in patients with adult spinal deformity.

## 5. Conclusions

While patients with poor preoperative mental health have a greater disease severity both before and after ASD surgery, they appear to experience comparable benefits from surgical intervention relative to their counterparts without mental health conditions. Recognizing and managing mental health may be useful in the preoperative management of ASD patients. Further prospective studies to further elucidate these associations are necessary.

## Figures and Tables

**Table 1:** Summary of included studies.

Study	Study Setting	Study Type	N	Mean Age	Gender (% Female)	Comorbidity Assessed	Measure of Mental Health	Outcome Measure	Follow-Up Time Point
Theologis et al., 2016 [11]	Multicenter	Retrospective	267	56	83.9	Depression	Self-Reported	ODI, SRS22, SF-36 PCS, Back Pain, Leg Pain	2 years
Elsamadicy et al., 2017 [25]	Single Center	Retrospective	923	61.25	63.9	Depression	Previous Diagnosis	Complications	Postoperative
Bakhsheshian et al., 2017 [10]	Multicenter	Retrospective	144	57.49	83.3	Mental Health	SF-36 MCS	ODI, SRS22, SF-36 PCS, SF-36 MCS, NRS Back, NRS Leg, Activity, Pain, Self-Image, Mental Health	2 years
Adogwa et al., 2017 [22]	Single Center	Retrospective	92	72.2	63.2	Depression	Koenig Depression Scale	VAS	6 months
Toombs et al., 2018 [38]	NIS	Retrospective	219975	58.7	67.6	Multiple Mental Health Disorders	ICD code	Major Medical Complications	Postoperative
Goh et al., 2018 [26]	Multicenter	Prospective	46	61.1		Psychiatric Condition (Depression or Anxiety)	Zung Depression and Anxiety Scale	SRS-22, PCS, MCS, RMDQ	2 years
Kyrölä et al., 2019 [29]	Single Center	Retrospective	79	64.3	72.2	Depression	Previous Diagnosis	SRS-30, ODI	5 years
Watanabe et al., 2020 [39]	Single Center	Retrospective	165	63	86.1	Mental Health	Previous Diagnosis	ODI, SRS-22	2 years
Hayashi et al., 2020 [27]	Multicenter	Retrospective	391	53.4	81.1	Mental Health	SF-36 MCS	SRS-22r SI Module	2 years
Yagi et al., 2021 [41]	Multicenter	Retrospective	159	64.6	94	Mental Health	SRS Mental Health Domain	SRS-22	2 years
Lafage et al., 2021 [30]	Multicenter	Retrospective	513	58.4	79	Mental Health	SF-36 MCS	ODI, SRS22, SF-36 PCS, SF-36 MCS	2 years
Morrissette et al., 2022 [33]	Single Center	Retrospective	100	44	74	Mental Health	SRS Mental Health Domain	SRS, ODI	2 years
Soroceanu et al., 2016 [36]	Multicenter	Retrospective	448	56.8	75	Depression	N/A	Complications	2 years
Diebo et al., 2018 [24]	Multicenter	Retrospective	6020	59	57.2	Multiple Mental Health Disorders	ICD Code	Readmission	2 years
Shah et al., 2019 [35]	PearlDiver	Retrospective	654	72.6	73.7	Depression/Anxiety	ICD Code	Complications	1 year
Lee et al., 2021 [31]	Single Center	Retrospective	175	52.6	72	Depression	N/A	Readmission	2 years
Williamson et al., 2022 [40]	Single Center	Retrospective	480		79	Depression	Previous Diagnosis	Complications	2 years
Zuckerman et al., 2022 [42]	Single Center	Retrospective	243	49.3	67	Depression/Anxiety	N/A	Postoperative Iatrogenic CM	2 years
Taliaferro et al., 2022 [37]	Multicenter	Retrospective	12641	61	69	Depression	ICD Code	Readmission	90 days
Lee et al., 2022 [32]	Single Center	Retrospective	227	50.5	67.8	Depression	N/A	Readmission	90 days
Arora et al., 2023 [23]	Single Center	Retrospective	355	66.9	62.5	Depression/Anxiety	Previous Diagnosis	Extended Length of Stay	Postoperative
Jamil et al., 2023 [28]	PearlDiver	Retrospective	3706		63.1	Depression	ICD Code	Complications	90 days
Onafowokan et al., 2024 [34]	Single Center	Retrospective	477	64.2		Depression	Previous Diagnosis	Complications	2 years
Agarwal et al., 2024 [12]	Multicenter	Retrospective	147	69	57.8	Depression	Self-Reported	ODI, SRS22, SF-36 PCS, SF-36 MCS, NRS Back, NRS Leg, EQ-5D, EQ-5D VAS	1 year

ODI-Oswestry Disability Index; SRS22-Scoliosis Research Society-22 Questionnaire; SF-36 PCS-Short Form-36 Physical Component Summary; SF-36 MCS-Short Form-36 Mental Component Summary; NRS Back-Numeric Rating Scale for Back Pain; NRS Leg-Numeric Rating Scale for Leg Pain; EQ-5D-EuroQol 5-Dimension Questionnaire; EQ-5D VAS-EuroQol 5-Dimension Visual Analog Scale; and RMDQ-Roland Morris Disability Questionnaire.

## Data Availability

Data can be made available upon reasonable request.

## Supplementary Materials

The following supporting information can be downloaded at: https://www.mdpi.com/article/10.3390/jcm14155516/s1, eAppendix 1. Study design and protocol as registered with International Platform of Registered Systematic Review and Meta-analysis Protocols (INPLASY) (registration number INPLASY202560113), eAppendix 2. Search Strategy, eAppendix 3. ROBINS-Iv2 Tool, eAppendix 4. Newcastle–Ottawa Scale for Assessment of Study Quality, eAppendix 5. Sensitivity analysis via Leave one out for A. HRQOL, B. Pain, C. Complications, D. Preoperative disease severity, and E. Postoperative disease severity, eAppendix 6. Funnel Plot for Detection of Publication Bias for A. HRQOL, B. Pain, C. Complications, D. Preoperative disease severity, and E. Postoperative disease severity eAppendix 7. Meta-Regression Analysis of Sources of Heterogeneity in Disability and Functional Outcomes, eAppendix 8. Meta-Regression Analysis of Sources of Heterogeneity in Pain Outcomes, eAppendix 9. Meta-Regression Analysis of Sources of Heterogeneity in Complications, eAppendix 10. Patient HRQOL stratified by A. overall mental health and B. Depression/Anxiety, eAppendix 11. Patient complications stratified by A. overall mental health and B. Depression/Anxiety, eAppendix 12. Strength of evidence assessment via the Grading of Recommendations, Assessment, Development, and Evaluations (GRADE) approach.

## Abbreviations

The following abbreviations are used in this manuscript:

ASD	Adult Spinal Deformity
HRQOL	Health Related Quality of Life
SMD	Standardized Mean Difference
PTSD	Post-traumatic Stress Disorder
