# Supplementary material for: The Association of Poor Preoperative Mental Health and Outcomes After Surgical Correction of Adult Spinal Deformity: A Systematic Review and Meta Analysis
Source: J Clin Med. 2025 Aug 5;14(15):5516. doi: 10.3390/jcm14155516 (PMC12347260; doi:10.3390/jcm14155516)
Supplement: Supplementary file 1 [file jcm-14-05516-s001.zip › jcm-3765681-supplementary.pdf]

<u>SUPPLEMENTARY DIGITAL CONTENT</u>

eAppendix 1. Study design and protocol as registered with International Platform of Registered Systematic Review and Meta-analysis Protocols (INPLASY) (registration number INPLASY202560113).

The Association of Poor Preoperative Mental Health and Outcomes after Surgical Correction of Adult Spinal Deformity: A Systematic Review and Meta Analysis

Introduction

Review question / Objective

To determine if preoperative mental health comorbidities are associated with poor surgical outcomes for adult spinal deformity (ASD).

Rationale

Adult Spinal Deformity (ASD) is pathologic malalignment of the spine and can result in significant loss of quality of life, loss of function, and morbidity.[1] Significantly, untreated ASD can often result in high rates of mental health comorbidities such as depression and self-image issues due to the deforming nature of these conditions.[2] Additionally, prolonged and chronic loss of function may also negatively affect mental health.[3]

Surgical intervention is highly effective method of treating ASD, resulting in improved quality of life and function for many. However, there has been increasing levels of evidence highlighting the significant role of preoperative mental health comorbidities in predicting post operative outcomes in both the cervical and lumbar spine populations.[4,5] Significantly, the role of mental health comorbidities in the ASD population, which may be disproportionately enriched in mental health comorbidities, has been suggested by several studies.[6-8] However, to our knowledge, no systematic review and meta-analysis has been conducted to ascertain the effects of pre-operative mental health comorbidities on surgical outcomes in patients with ASD.

We seek to perform a systematic review of the literature for any prospective, retrospective, or RCT studies that investigated the effect of pre-operative mental health comorbidities, including but not limited to overall measures of mental health, depression,

anxiety, bipolar, and PTSD, on surgical outcomes for ASD and to perform a meta-analysis of these results. In doing so, we hope to better understand the true effect of mental health comorbidities on ASD surgical outcomes.

Condition being studied

ASD is a common pathology that is associated with significant loss of quality of life and function. Surgical interventions for the correction of these deformities are highly effective and commonly performed. However, the role that mental health comorbidities play in outcomes remain unclear.

Participant or population

Adult patients (>18) with adult spinal deformity and underwent surgical treatment

Intervention

Pre-operative mental health comorbidities

Comparator

Patients with preoperative mental health comorbidities compared to those without

Study designs to be included

Observational studies, RCTs

Information sources

Medline, Embase, Web of Science, Scopus

Main outcome(s)

Odds Ratio for Complications

Odds Ratio for Readmissions

Change in PROs after operation

Methods

Study Design

Systematic review of the literature and meta-analysis using aggregate-level data with adherence to the PRISMA checklist guideline

Inclusion/Exclusion Criteria

Inclusion:

Study inclusion criteria:

- All observation studies from inception to April 2025

Patient/participant inclusion criteria:

- Adult patients (>18) undergoing surgery for correction of ASD

Exclusions:

Study exclusion criteria:

Exclusion: Case series, case reports, brief reports, pilot reports, opinion pieces, theses, conference proceedings, letters and commentaries, editorials, meta-analysis and reviews, surgical technique papers, abstracts, conference proceedings, and non-English language articles without available translations.

Information sources:

Medline, Embase, Web of Science, and Scopus

Search Strategy:

Medline/Pubmed:

(

"Mental Disorders"[Mesh] OR "Mental Health"[Mesh] OR "Depression"[Mesh] OR "Anxiety Disorders"[Mesh]
OR "Bipolar Disorder"[Mesh] OR "Stress Disorders, Post-Traumatic"[Mesh] OR "Obsessive-Compulsive Disorder"[Mesh]
OR "Schizophrenia"[Mesh] OR "Substance-Related Disorders"[Mesh] OR "Personality Disorders"[Mesh]
OR "Somatoform Disorders"[Mesh] OR depression[tiab] OR depressive[tiab] OR "major depressive disorder"[tiab]

OR anxiety[tiab] OR "generalized anxiety disorder"[tiab] OR "panic disorder"[tiab]
OR "post-traumatic stress disorder"[tiab] OR PTSD[tiab] OR bipolar[tiab] OR "bipolar disorder"[tiab]
OR "mood disorder"[tiab] OR schizophrenia[tiab] OR psychosis[tiab] OR "psychotic disorder"[tiab]
OR "substance use disorder"[tiab] OR "alcohol use disorder"[tiab] OR "drug use disorder"[tiab]
OR "eating disorder"[tiab] OR "anorexia nervosa"[tiab] OR "bulimia nervosa"[tiab]
OR "personality disorder"[tiab] OR "adjustment disorder"[tiab] OR "somatic symptom disorder"[tiab]
OR "conversion disorder"[tiab] OR "obsessive-compulsive disorder"[tiab] OR OCD[tiab]
OR "psychological distress"[tiab] OR "mental illness"[tiab] OR "psychiatric disorder"[tiab]
)
AND
(
"Spinal Curvatures"[Mesh] OR "Scoliosis"[Mesh] OR "Kyphosis"[Mesh]
OR scoliosis[tiab] OR kyphosis[tiab] OR "adult spinal deformity"[tiab]
OR "thoracolumbar deformity"[tiab] OR "sagittal imbalance"[tiab]
OR "spinal malalignment"[tiab] OR "spinal deformity"[tiab]
)

N=1371

Embase Search Terms:

('depression' OR 'anxiety disorder' OR 'bipolar disorder' OR 'posttraumatic stress disorder' OR 'obsessive compulsive disorder' OR 'schizophrenia' OR depression:ti,ab OR depressive:ti,ab OR 'major depressive disorder':ti,ab OR anxiety:ti,ab OR 'generalized anxiety disorder':ti,ab OR 'panic disorder':ti,ab OR 'post-traumatic stress disorder':ti,ab OR ptsd:ti,ab OR bipolar:ti,ab OR 'bipolar disorder':ti,ab OR schizophrenia:ti,ab) AND ('scoliosis' OR 'kyphosis' OR scoliosis:ti,ab OR kyphosis:ti,ab OR 'adult spinal

deformity':ti,ab OR 'thoracolumbar deformity':ti,ab OR 'sagittal imbalance':ti,ab OR 'spinal malalignment':ti,ab OR 'spinal deformity':ti,ab)

N=1486

Web of Science Search Terms:

TS=(

depression OR depressive OR "major depressive disorder"

OR anxiety OR "generalized anxiety disorder" OR "panic disorder"

OR "post-traumatic stress disorder" OR PTSD OR bipolar OR "bipolar disorder"

OR "mood disorder" OR schizophrenia OR psychosis OR "psychotic disorder"

OR "substance use disorder" OR "alcohol use disorder" OR "drug use disorder"

OR "eating disorder" OR "anorexia nervosa" OR "bulimia nervosa"

OR "personality disorder" OR "adjustment disorder" OR "somatic symptom disorder"

OR "conversion disorder" OR "obsessive compulsive disorder" OR OCD

OR "psychological distress" OR "mental illness" OR "psychiatric disorder"

OR "mental health"

)

AND

TS=(

"spinal curvature" OR scoliosis OR kyphosis

OR "adult spinal deformity" OR "thoracolumbar deformity"

OR "sagittal imbalance" OR "spinal malalignment" OR "spinal deformity"

)

N=2174

Scopus:

(

TITLE-ABS-KEY(depression) OR TITLE-ABS-KEY(depressive) OR TITLE-ABS-KEY("major depressive disorder")

OR TITLE-ABS-KEY(anxiety) OR TITLE-ABS-KEY("generalized anxiety disorder") OR TITLE-ABS-KEY("panic disorder")

OR TITLE-ABS-KEY("post-traumatic stress disorder") OR TITLE-ABS-KEY(PTSD) OR TITLE-ABS-KEY(bipolar)

OR TITLE-ABS-KEY("bipolar disorder") OR TITLE-ABS-KEY("mood disorder") OR TITLE-ABS-KEY(schizophrenia)

OR TITLE-ABS-KEY(psychosis) OR TITLE-ABS-KEY("psychotic disorder") OR TITLE-ABS-KEY("substance use disorder")

OR TITLE-ABS-KEY("alcohol use disorder") OR TITLE-ABS-KEY("drug use disorder")

OR TITLE-ABS-KEY("eating disorder") OR TITLE-ABS-KEY("anorexia nervosa")

OR TITLE-ABS-KEY("bulimia nervosa") OR TITLE-ABS-KEY("personality disorder")

OR TITLE-ABS-KEY("adjustment disorder") OR TITLE-ABS-KEY("somatic symptom disorder")

OR TITLE-ABS-KEY("conversion disorder") OR TITLE-ABS-KEY("obsessive compulsive disorder")

OR TITLE-ABS-KEY(OCD) OR TITLE-ABS-KEY("psychological distress")

OR TITLE-ABS-KEY("mental illness") OR TITLE-ABS-KEY("psychiatric disorder")

OR TITLE-ABS-KEY("mental health")

)

AND

(

TITLE-ABS-KEY("spinal curvature") OR TITLE-ABS-KEY("scoliosis") OR TITLE-ABS-KEY("kyphosis")

OR TITLE-ABS-KEY("adult spinal deformity") OR TITLE-ABS-KEY("thoracolumbar deformity")
OR TITLE-ABS-KEY("sagittal imbalance") OR TITLE-ABS-KEY("spinal malalignment")
OR TITLE-ABS-KEY("spinal deformity")
)

N=916

Data Items for extraction:

Study: (First author name followed by *et al.*)

Year of publication

Effect size for each pre-defined outcome variable

Upper limit CI for each pre-defined outcome variable

Lower limit CI for each pre-defined outcome variable

Study size

Outcome reported

Mental Health Comorbidity Assessed

Method of mental health comorbidity assessment

Demographic and patient enrollment characteristics

Metadata

- Journal name where study was published.
- Year of publication

Outcomes and Prioritization

- Primary outcome:

    - Change in HRQOL after surgery
    - Complications
- Secondary outcomes:
    - Pre and Post operative disease Severity

Risk of bias of individual studies

Risk of bias will be assessed at the study level:

- The ROBINS-I v2 tool will be utilized
- A funnel plot using Egger tests will be used to assess publication bias
- We will also use the Newcastle-Ottawa Scale (NOS) to assess study quality

Data synthesis:

Due to predicted variability in patient selection among the studies, we will utilize a random effects model with restricted maximum-likelihood estimation for the meta-analysis and data synthesis. Study heterogeneity will be assessed using the inconsistency index (I^2). Meta-regression will be utilized to assess reasons for heterogeneity.

eAppendix 2. Search Strategy

Medline/Pubmed:

(

"Mental Disorders"[Mesh] OR "Mental Health"[Mesh] OR "Depression"[Mesh] OR "Anxiety Disorders"[Mesh]
OR "Bipolar Disorder"[Mesh] OR "Stress Disorders, Post-Traumatic"[Mesh] OR "Obsessive-Compulsive Disorder"[Mesh]
OR "Schizophrenia"[Mesh] OR "Substance-Related Disorders"[Mesh] OR "Personality Disorders"[Mesh]
OR "Somatoform Disorders"[Mesh] OR depression[tiab] OR depressive[tiab] OR "major depressive disorder"[tiab]

OR anxiety[tiab] OR "generalized anxiety disorder"[tiab] OR "panic disorder"[tiab]

OR "post-traumatic stress disorder"[tiab] OR PTSD[tiab] OR bipolar[tiab] OR "bipolar disorder"[tiab]

OR "mood disorder"[tiab] OR schizophrenia[tiab] OR psychosis[tiab] OR "psychotic disorder"[tiab]

OR "substance use disorder"[tiab] OR "alcohol use disorder"[tiab] OR "drug use disorder"[tiab]

OR "eating disorder"[tiab] OR "anorexia nervosa"[tiab] OR "bulimia nervosa"[tiab]

OR "personality disorder"[tiab] OR "adjustment disorder"[tiab] OR "somatic symptom disorder"[tiab]

OR "conversion disorder"[tiab] OR "obsessive-compulsive disorder"[tiab] OR OCD[tiab]

OR "psychological distress"[tiab] OR "mental illness"[tiab] OR "psychiatric disorder"[tiab]

)

AND

(

"Spinal Curvatures"[Mesh] OR "Scoliosis"[Mesh] OR "Kyphosis"[Mesh]

OR scoliosis[tiab] OR kyphosis[tiab] OR "adult spinal deformity"[tiab]

OR "thoracolumbar deformity"[tiab] OR "sagittal imbalance"[tiab]

OR "spinal malalignment"[tiab] OR "spinal deformity"[tiab]

)

N=1371

Embase Search Terms:

('depression' OR 'anxiety disorder' OR 'bipolar disorder' OR 'posttraumatic stress disorder' OR 'obsessive compulsive disorder' OR 'schizophrenia' OR depression:ti,ab OR depressive:ti,ab OR 'major depressive disorder':ti,ab OR anxiety:ti,ab OR 'generalized anxiety disorder':ti,ab OR 'panic disorder':ti,ab OR 'post-traumatic stress disorder':ti,ab OR ptsd:ti,ab OR bipolar:ti,ab OR 'bipolar disorder':ti,ab OR schizophrenia:ti,ab) AND ('scoliosis' OR 'kyphosis' OR scoliosis:ti,ab OR kyphosis:ti,ab OR 'adult spinal

deformity':ti,ab OR 'thoracolumbar deformity':ti,ab OR 'sagittal imbalance':ti,ab OR 'spinal malalignment':ti,ab OR 'spinal deformity':ti,ab)

N=1486

Web of Science Search Terms:

TS=(

depression OR depressive OR "major depressive disorder"

OR anxiety OR "generalized anxiety disorder" OR "panic disorder"

OR "post-traumatic stress disorder" OR PTSD OR bipolar OR "bipolar disorder"

OR "mood disorder" OR schizophrenia OR psychosis OR "psychotic disorder"

OR "substance use disorder" OR "alcohol use disorder" OR "drug use disorder"

OR "eating disorder" OR "anorexia nervosa" OR "bulimia nervosa"

OR "personality disorder" OR "adjustment disorder" OR "somatic symptom disorder"

OR "conversion disorder" OR "obsessive compulsive disorder" OR OCD

OR "psychological distress" OR "mental illness" OR "psychiatric disorder"

OR "mental health"

)

AND

TS=(

"spinal curvature" OR scoliosis OR kyphosis

OR "adult spinal deformity" OR "thoracolumbar deformity"

OR "sagittal imbalance" OR "spinal malalignment" OR "spinal deformity"

)

N=2174

Scopus:

(

TITLE-ABS-KEY(depression) OR TITLE-ABS-KEY(depressive) OR TITLE-ABS-KEY("major depressive disorder")

OR TITLE-ABS-KEY(anxiety) OR TITLE-ABS-KEY("generalized anxiety disorder") OR TITLE-ABS-KEY("panic disorder")

OR TITLE-ABS-KEY("post-traumatic stress disorder") OR TITLE-ABS-KEY(PTSD) OR TITLE-ABS-KEY(bipolar)

OR TITLE-ABS-KEY("bipolar disorder") OR TITLE-ABS-KEY("mood disorder") OR TITLE-ABS-KEY(schizophrenia)

OR TITLE-ABS-KEY(psychosis) OR TITLE-ABS-KEY("psychotic disorder") OR TITLE-ABS-KEY("substance use disorder")

OR TITLE-ABS-KEY("alcohol use disorder") OR TITLE-ABS-KEY("drug use disorder")

OR TITLE-ABS-KEY("eating disorder") OR TITLE-ABS-KEY("anorexia nervosa")

OR TITLE-ABS-KEY("bulimia nervosa") OR TITLE-ABS-KEY("personality disorder")

OR TITLE-ABS-KEY("adjustment disorder") OR TITLE-ABS-KEY("somatic symptom disorder")

OR TITLE-ABS-KEY("conversion disorder") OR TITLE-ABS-KEY("obsessive compulsive disorder")

OR TITLE-ABS-KEY(OCD) OR TITLE-ABS-KEY("psychological distress")

OR TITLE-ABS-KEY("mental illness") OR TITLE-ABS-KEY("psychiatric disorder")

OR TITLE-ABS-KEY("mental health")

)

AND

(

TITLE-ABS-KEY("spinal curvature") OR TITLE-ABS-KEY("scoliosis") OR TITLE-ABS-KEY("kyphosis")

OR TITLE-ABS-KEY("adult spinal deformity") OR TITLE-ABS-KEY("thoracolumbar deformity")
OR TITLE-ABS-KEY("sagittal imbalance") OR TITLE-ABS-KEY("spinal malalignment")
OR TITLE-ABS-KEY("spinal deformity")
)

N=916

eAppendix 3. ROBINS-Iv2 Tool

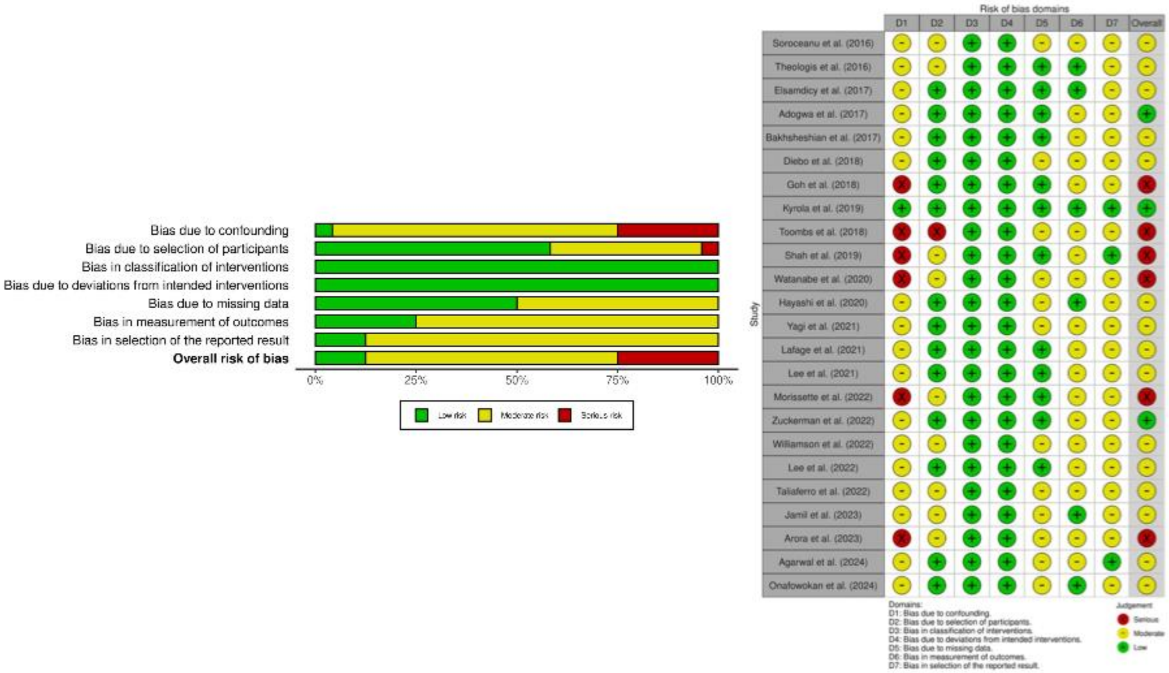

eAppendix 4. Newcastle Ottawa Scale for Assessment of Study Quality

Author	Year	Selection				Comparability	Outcome			Total score	Quality
		Adequate definition of exposed cohort	Selection of the non-exposed cohort	Ascertainment of Exposure	Demonstration that outcome of interst was not present at start of study	Control for important or additional factors	Assessment of Outcome	Follow-up Length (>2 years for PROMs, as appropriate for complications)	Follow-up rate		
Theologis et al. 2016	2016	⋆	⋆	⋆	⋆	⋆⋆	⋆	⋆	⋆	9	Good
Elsamadicy et al. 2017	2017	⋆	⋆	⋆	⋆	⋆⋆	⋆	⋆	⋆	9	Good
Bakhsheshian et al. 2017	2017	⋆	⋆	⋆	⋆	⋆⋆	⋆	⋆	⋆	9	Good
Adogwa et al. 2017	2017	⋆	⋆	⋆	⋆		⋆		⋆	6	Poor
Toombs et al. 2018	2018	⋆	⋆	⋆	⋆	⋆⋆	⋆	⋆	⋆	9	Good
Goh et al. 2018	2018	⋆	⋆	⋆	⋆	⋆⋆	⋆	⋆	⋆	9	Good

Kyrölä et al. 2019	2019	★	★	★	★	★★	★	★	★	9	Good
Watanabe et al. 2020	2020	★	★	★	★		★	★	★	8	Poor
Hayashi et al. 2020	2020	★	★	★	★	★★	★	★	★	9	Good
Yagi et al. 2021	2021	★	★	★	★	★★	★	★	★	9	Good
Lafage et al. 2021	2021	★	★	★	★	★★	★	★	★	9	Good
Morrissette et al. 2022	2022	★	★	★	★	★★	★	★	★	9	Good
Soroceanu et al. 2016	2016	★	★		★	★★	★	★	★	8	Good
Diebo et al. 2018	2018	★	★	★	★	★★	★	★	★	9	Good
Shah et al. 2019	2019	★	★	★	★	★★	★	★	★	9	Good
Lee et al. 2021	2021	★	★		★	★★	★	★	★	8	Good
Morisette et al. 2022	2022	★	★	★	★	★★	★	★	★	9	Good

Zuckerman et al. 2022	2022	⋆	⋆		⋆	⋆⋆	⋆	⋆	⋆	8	Good
Taliaferro et al. 2022	2022	⋆	⋆	⋆	⋆	⋆⋆	⋆	⋆	⋆	9	Good
Lee et al. 2022	2022	⋆	⋆		⋆	⋆⋆	⋆	⋆	⋆	8	Good
Aurora et al. 2023	2023	⋆	⋆	⋆	⋆	⋆⋆	⋆	⋆	⋆	9	Good
Jamil et al 2023	2023	⋆	⋆	⋆	⋆	⋆⋆	⋆	⋆	⋆	9	Good
Onafowokan et al. 2024	2024	⋆	⋆	⋆	⋆	⋆⋆	⋆	⋆	⋆	9	Good
Agarwal et al. 2024	2024	⋆	⋆	⋆	⋆	⋆⋆	⋆		⋆	8	Good

eAppendix 5. Sensitivity analysis via Leave one out for **A**. HRQOL, **B.** Pain, **C.** Complications, **D.** Preoperative disease severity, and **E.** Post operative disease severity

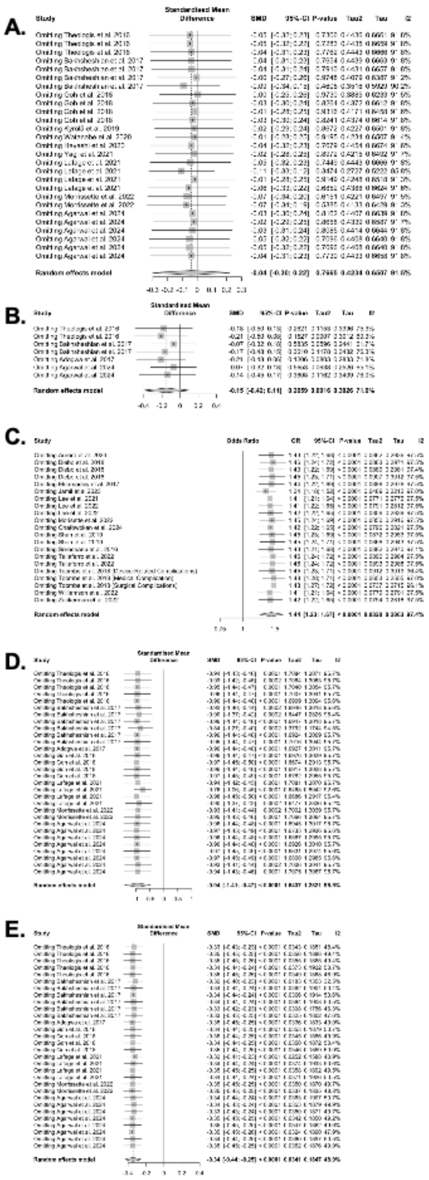
A.
B.
C.
D.
E.
Study
Standardised Mean Difference
SMD
95%-CI
P-value
Tau2
Tau
I2
Odds Ratio
OR
Random effects model

eAppendix 6. Funnel Plot for Detection of Publication Bias for **A.** HRQOL, **B.** Pain, **C.** Complications, **D.** Preoperative disease severity, and **E.** Post operative disease severity

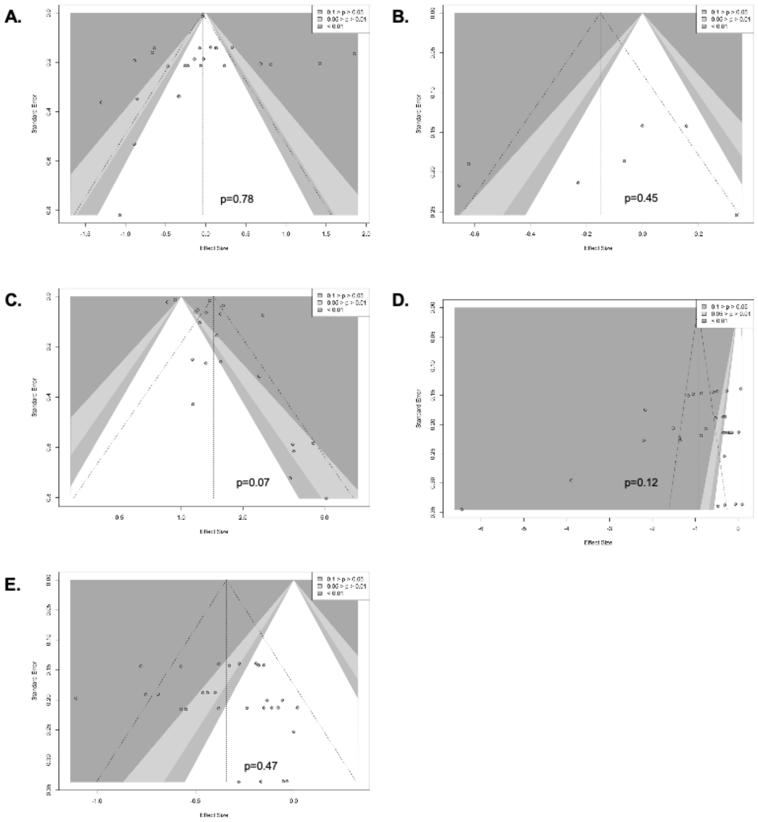

eAppendix 7. Meta-Regression Analysis of Sources of Heterogeneity in Disability and Functional Outcomes

Disability and Function	**exp(Beta)**	**95% CI**	**p-value**
intercept	0	0.00, 0.00	0.007
Total N	1	1.00, 1.00	0.045
Mean Age	1.36	1.08, 1.71	0.008
% Gender Female	1.17	1.04, 1.32	0.007
Outcome measure: EQ-5D VAS	1.28	0.35, 4.76	0.7
Outcome measure: ODI	1.31	0.44, 3.95	0.6
Outcome measure: SF-36 MCS	4.56	1.47, 14.2	0.009
Outcome measure: SF-36 PCS	0.92	0.30, 2.81	0.9
Outcome measure: SRS-22	1.38	0.46, 4.15	0.6
Outcome measure: SRS-30/ODI	0	0.00, 0.01	0.002
Comorbidity Assessed: Overall Mental Health	0	0.00, 0.00	0.002
Method of Comorbidity Assessment: SF-36 MCS	1,222,060	174, 8,568,451,438	0.002
Method of Comorbidity Assessment: SRS mental health domain	10,948	29.7, 4,038,428	0.002
Study Setting: Single Center	81,760	59.4, 112,448,240	0.002

Abbreviation: CI = Confidence Interval

R^2 = 55.6

eAppendix 8. Meta-Regression Analysis of Sources of Heterogeneity in Pain Outcomes

Pain	exp(Beta)	95% CI	p-value
intercept	0	0.00, 0.03	0.015
Total N	1.01	1.00, 1.01	0.043
Mean Age	1.2	1.03, 1.40	0.018
% Gender Female	1.09	1.01, 1.18	0.021

Abbreviation: CI = Confidence Interval

R^2=55.5

eAppendix 9. Meta-Regression Analysis of Sources of Heterogeneity in Complications

Complications	exp(Beta)	95% CI	p-value
intercept	1.51	0.02, 109	0.8
Total N	1	1.00, 1.00	>0.9
% Gender Female	1	0.95, 1.04	>0.9
Study Setting: NIS	2.45	0.00, 4,610,455,702,905	>0.9
Study Setting: PearlDiver	1.7	0.44, 6.65	0.4
Study Setting: Single Center	2.77	1.06, 7.20	0.037
Comorbidity Assessed: Depression/Anxiety	0.64	0.40, 1.03	0.067
Comorbidity Assessed: Mental Health	0.32	0.07, 1.42	0.13
Comorbidity Assessed: Multiple Mental Health Disorders	1.03	0.29, 3.62	>0.9
Method of Comorbidity Assessment: N/A	1.21	0.30, 4.88	0.8
Method of Comorbidity Assessment: Previous Diagnosis	0.52	0.15, 1.78	0.3

Abbreviation: CI = Confidence Interval

R^2=55.5

eAppendix 10. Patient HRQOL stratified by A. overall mental health and B. Depression/Anxiety

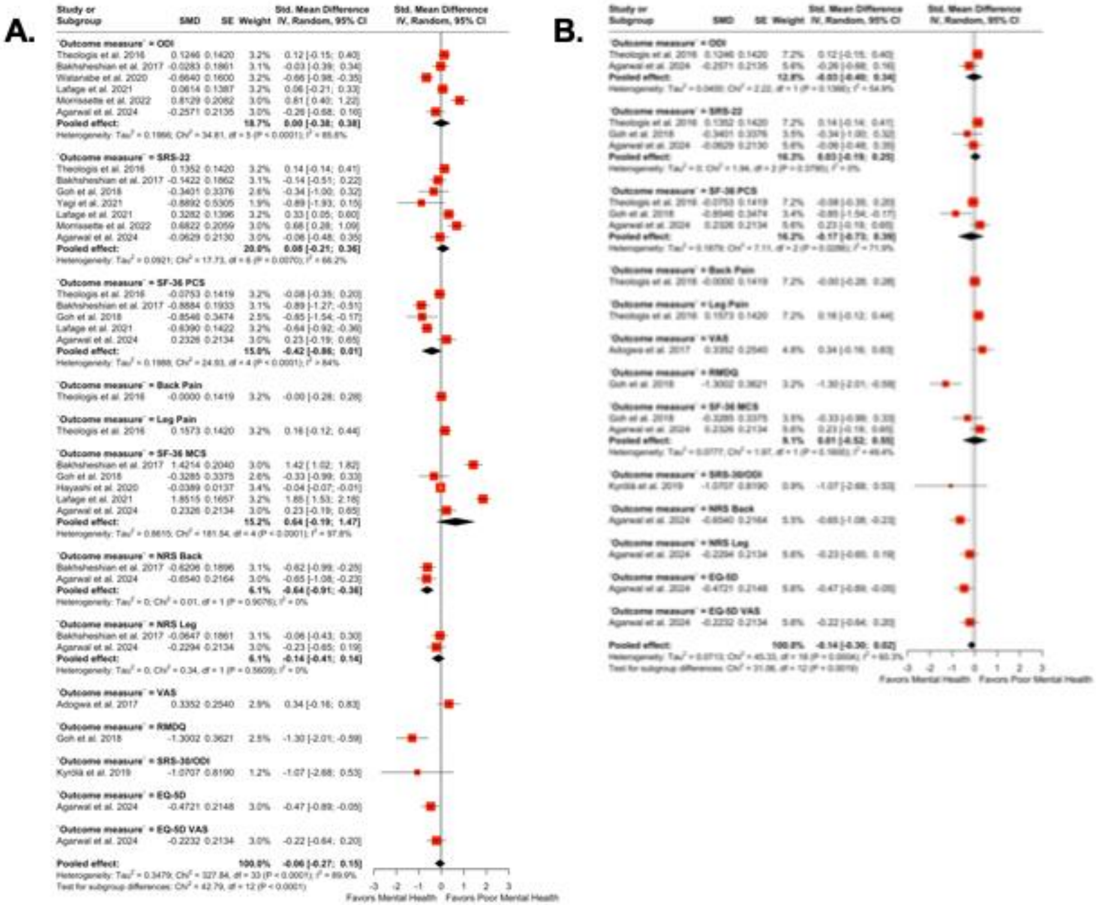

eAppendix 11. Patient complications stratified by A. overall mental health and B. Depression/Anxiety

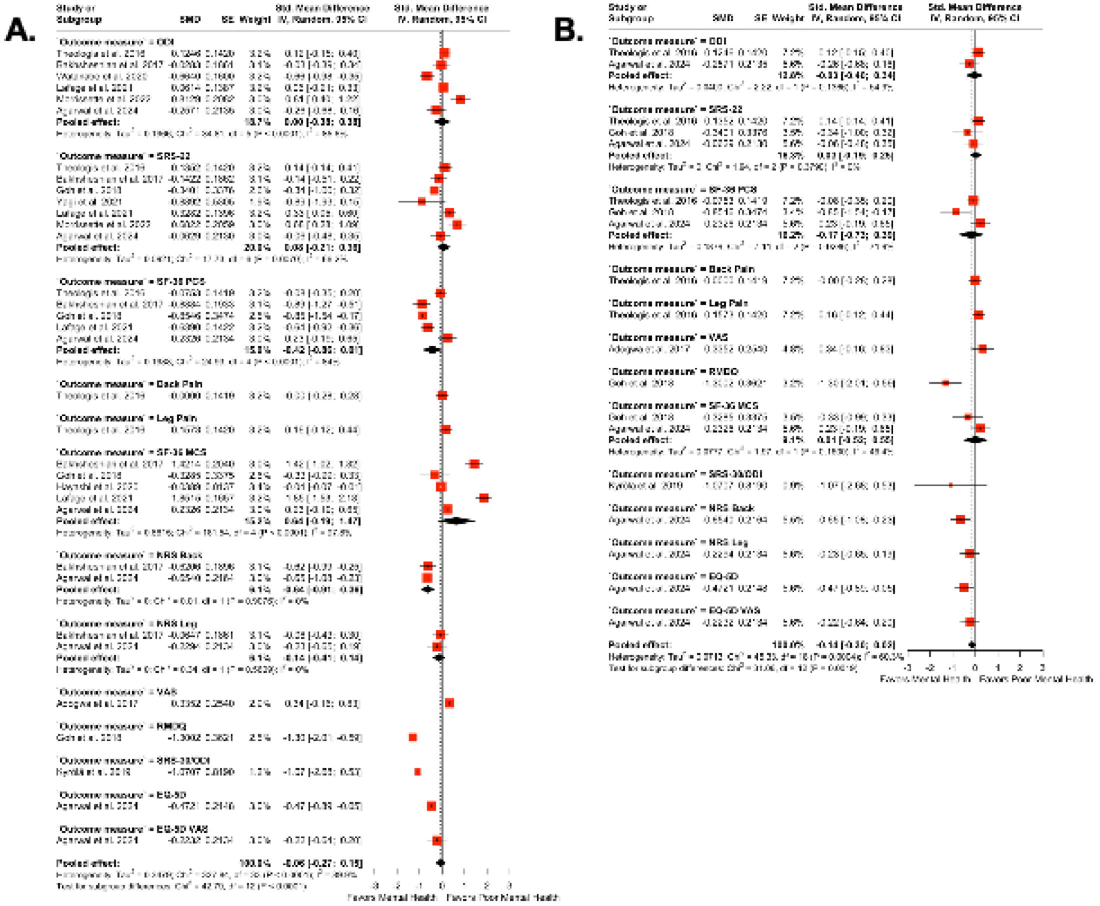

eAppendix 12. Strength of evidence assessment via the Grading of Recommendations, Assessment, Development, and Evaluations (GRADE) approach

Certainty Assessment							Effect	
No. of studies	Study Design	Risk of bias	Inconsistency	Indirectness	Imprecision	Other Considerations	Effect Size (95%CI)	Certainty
**Post-operative Improvement (Follow-up: range 2 years to 5 years)**								
11	Observation, non-randomized	not serious	serious[a]	not serious	not serious	no publication bias	Function SMD: -0.04 (95%CI -0.30 to 0.22), Pain SMD: -0.15 (95%CI -0.42 to 0.11)	⊕⊕⊕◯ Moderate
**Post-operative Complications**								
13	Observation, non-randomized	not serious	serious[a]	not serious	not serious	no publication bias	OR 1.44 (95%CI 1.23 to 1.67)	⊕⊕⊕◯ Moderate
**Pre-operative Disease Severity**								
11	Observation, non-randomized	not serious	serious[a]	not serious	not serious	no publication bias	SMD -0.94 (95%CI -1.41 to -0.47)	⊕⊕⊕◯ Moderate
**Post-operative Disease Severity**								
11	Observation, non-randomized	not serious	serious[a]	not serious	not serious	no publication bias	SMD -0.34 (95%CI -0.44 to -0.25)	⊕⊕⊕◯ Moderate
[a] inconsistency between exposure definitions								
